# High-Frequency Repetitive Sensory Stimulation as Intervention to Improve Sensory Loss in Patients with Complex Regional Pain Syndrome I

**DOI:** 10.3389/fneur.2015.00242

**Published:** 2015-11-17

**Authors:** Marianne David, Hubert R. Dinse, Tina Mainka, Martin Tegenthoff, Christoph Maier

**Affiliations:** ^1^Department of Pain Medicine, Berufsgenossenschaftliches Universitätsklinikum Bergmannsheil, Ruhr-University Bochum, Bochum, Germany; ^2^Neural Plasticity Laboratory, Institute for Neuroinformatics, Ruhr-University Bochum, Bochum, Germany; ^3^Department of Neurology, Berufsgenossenschaftliches Universitätsklinikum Bergmannsheil, Ruhr-University Bochum, Bochum, Germany; ^4^Department of Neurology, University Medical Center Hamburg-Eppendorf, Hamburg, Germany

**Keywords:** complex regional pain syndrome, CRPS, repetitive sensory stimulation, rSS, cortical reorganization, tactile performance

## Abstract

Achieving perceptual gains in healthy individuals or facilitating rehabilitation in patients is generally considered to require intense training to engage neuronal plasticity mechanisms. Recent work, however, suggested that beneficial outcome similar to training can be effectively acquired by a complementary approach in which the learning occurs in response to mere exposure to repetitive sensory stimulation (rSS). For example, high-frequency repetitive sensory stimulation (HF-rSS) enhances tactile performance and induces cortical reorganization in healthy subjects and patients after stroke. Patients with complex regional pain syndrome (CRPS) show impaired tactile performance associated with shrinkage of cortical maps. We here investigated the feasibility and efficacy of HF-rSS, and low-frequency rSS (LF-rSS) to enhance tactile performance and reduce pain intensity in 20 patients with CRPS type I. Intermittent high- or low-frequency electrical stimuli were applied for 45 min/day to all fingertips of the affected hand for 5 days. Main outcome measures were spatial two-point-discrimination thresholds and mechanical detection thresholds measured on the tip of the index finger bilaterally. Secondary endpoint was current pain intensity. All measures were assessed before and on day 5 after the last stimulation session. HF-rSS applied in 16 patients improved tactile discrimination on the affected hand significantly without changes contralaterally. Current pain intensity remained unchanged on average, but decreased in four patients by ≥30%. This limited pain relief might be due to the short stimulation period of 5 days only. In contrast, after LF-rSS, tactile discrimination was impaired in all four patients, while detection thresholds and pain were not affected. Our data suggest that HF-rSS could be used as a novel approach in CRPS treatment to improve sensory loss. Longer treatment periods might be required to induce consistent pain relief.

## Introduction

The complex regional pain syndrome (CRPS) is a chronic pain syndrome, which is characterized by sensory, autonomic, and motor disturbances (1, 2). It is subdivided into type I without and type II with nerve lesion. In the development and maintenance of CRPS inflammatory processes, particularly at the beginning of the disease (3–5), vasomotor dysfunction (6, 7) and maladaptive cortical reorganization (8–10) mainly in later stages of the disease seem to play key roles. Aside from pharmacological treatment, rehabilitation based on concepts to induce neuroplasticity (11), such as sensory training, mirror therapy, or graded motor learning, are currently used to improve the sensorimotor limb function and to reduce pain (9, 12–16).

Changes of tactile abilities can be reliably induced not only by training and practice but also by brief, training-independent sensory learning through repetitive somatosensory stimulation (rSS) (17–19). From several studies in healthy young adult and elderly human subjects, it is known that rSS applied to the fingertips improves tactile acuity paralleled by plastic reorganizational changes within the primary and secondary somatosensory cortex (20–25). Translating protocols used in long-term potentiation (LTP), or long-term depression (LTD) studies to sensory stimulation experiments in humans, it could be shown that high-frequency rSS (HF-rSS) improved tactile acuity, while in contrast the application of low-frequency rSS (LF-rSS) lead to impaired tactile discrimination performance (26). Recently, it has been shown that long-term HF-rSS is effective to treat sensory loss and to improve motor performance after stroke (27, 28). The advantage of HF-rss over conventional neurorehabilitative approaches is its passive character, which allows improving sensorimotor function without task specific training, which is often complicated in patients with sensory deficits. Since, among other deficits, patients with CRPS show reduced tactile discrimination performance in combination with a shrunken cortical representation of the affected hand (9, 12, 29–33), rSS might be a promising intervention for an additional non-pharmacological concept in the treatment of CRPS.

A recent investigation demonstrated enhanced cortical excitability of the somatosensory and motor cortex in patients with CPRS (34, 35). Moreover, the occurrence of paradoxical stimulation effects after repetitive transcranial magnetic stimulation (rTMS) in other pathological states with cortical excitability changes, such as migraine, is well known (36, 37). Bearing this in mind, we also investigated whether in CRPS patients a LF-rSS protocol might, in contrast to healthy subjects, lead to improvement of tactile performance.

We therefore here explored the feasibility and efficacy of both a high-frequency rSS (HF-rSS) and a low-frequency rSS (LF-rSS) protocol over 45 min on five consecutive days to all fingertips of the affected hand in patients with CRPS. Primary outcome criteria were the two-point-discrimination (2PD) threshold and the mechanical detection threshold (MDT) of the index finger as measures of tactile performance. As additional secondary endpoint the potential impact on the current pain intensity was investigated.

## Materials and Methods

### Patients

The study was approved by the Ethics Committee of the Ruhr-University Bochum, Germany (4236-12) and was performed in accordance with the Declaration of Helsinki from October 2008. All patients gave their written informed consent. A total of 26 consecutive in-patients of the Department of Pain Medicine of the University Hospital Bergmannsheil Bochum who fulfilled the Budapest diagnostic criteria for CRPS (2) were recruited. An increased periarticular tracer uptake in the mineralization phase of a 3-phase bone scintigraphy served as additional positive diagnostic criterion (38). Exclusion criteria were CRPS type II, affecting the median or ulnar nerve. One patient with a lesion of the superficial branch of the radial nerve, innervating only the dorsum of the hand but not the palmar fingertips, was included. Further exclusion criteria were increasing pain during the intervention and withdrawal of consent or changes in the medication during the period of investigation.

A total of six patients were excluded (CRPS II: *n* = 5; medication change: *n* = 1). After inclusion in the study, prior to any intervention, a detailed history was taken and each patient underwent a physical examination with focus on assessment of the Budapest Criteria (2). Current pain intensity at rest as well as the average pain intensity during the last 4 weeks was rated by the patient on an 11-point numerical rating scale (NRS, 0–10). The distance between the tip of the middle finger and the palm was measured while the patient was asked to make a fist. Handedness was assessed by the Edinburgh handedness inventory (39). The clinical data of each of these 20 patients can be found in Table 1. The mean age of all patients who were included was 55.2 ± 2.1 years, mean disease duration 8.7 ± 5.8 months, mean current pain rating 3.5 ± 0.5 and mean average pain rating over the last 4 weeks 5.8 ± 2.0. All but two of the patients reported pain at rest before the start of the treatment.

**Table 1:** **Patients characteristics**.

Patient	Age (years)	Gender	Handedness	Affected hand	Inciting event	With/without surgery before onset	Disease duration (months)	Positive scintigraphy	Finger-palm-distance (cm)	Current pain^a^	Average pain (last 4 weeks)^a^	Sensory signs	Vasomotor signs	Sudormotor signs/edema	Motor/trophic signs	Current medication
**Stimulation with high-frequency pulses (hf-rss)**																
1	49	F	R	R	Fract.	−	9	+	3	7	7	+	+	+	+	Metamizol, AC, TCA
2	46	M	R	R	Fract.	+	5	+	0	0	7	+	+	+	+	Metamizol
3	57	F	R	L	Surg.	+	12	+	0	5	6	+	−	+	+	Metamizol, AC
4^b^	28	F	R	L	Trau.	+	4	n/a	11	3	5	+	+	−	+	Metamizol, AC, Opioids-III
5	59	F	R	R	Trau.	+	6	+	4	3	−	+	−	−	+	Opioids-II, NSAID
6	70	F	R	R	Surg.	+	11	+	5	3	4	+	+	+	+	Paracetamol
7	58	F	R	L	Fract.	−	12	n/s	5	5	5	+	−	−	+	Metamizol
8	58	M	R	L	Surg.	+	4	+	4	1	6	+	−	+	+	Metamizol, AC, Opioids-III, NSAID
9	60	F	R	L	Fract.	+	2	+	5	1	10	+	+	+	+	Metamizol, NSAID
10^c^	60	F	R	R	Fract.	+	15	+	4	4	6	+	+	+	+	AC, Opioids-III
11	63	F	R	L	Fract.	−	5	+	3.5	7	8	+	−	+	+	Metamizol, SNRI
12	52	M	R	R	Fract.	−	14	n/s	2.5	6	3	+	+	+	+	NSAID
13	52	M	R	L	Fract.	+	27	+	11	5	5	+	+	+	+	Metamizol, AC, Flupirtin
14	51	M	R	L	Fract.	−	4	+	6	3	4	+	+	+	+	NSAID, TCA
15	60	M	R	R	Fract.	+	8	+	2	2	9	+	+	+	+	Metamizol, AC
16^d^	41	M	R	R	Trau.	+	5	+	2	2	2	+	−	+	+	Metamizol
**Pilot testing with low-frequency pulses (lf-rss)**																
17	71	M	R	L	Fract.	+	6	+	6	6	6	+	+	+	+	AC, Opioids-III, NSAID
18	53	M	R	L	Fract.	−	12	+	1	4	5	+	−	+	+	Opioids-II, NSAID
19	58	F	R	R	Surg.	+	9	−	3	3	7	+	−	+	+	AC, Opioids-III, NSAID, Flupirtin
20	58	M	L	L	Fract.	+	4	+	3	0	5	+	+	+	+	NSAID

*F, female; M, male; R, right; L, left; Fract, fracture; surg, handsurgery for preceding disease (e.g., M. Dupuytren, rhizarthritis); trau, soft tissue trauma (e.g., incision wounds, crush injury), bone metabolism; +, increased periarticular tracer uptake in 3-phase bone scintigraphy; n/a, not available; n/s, later than 8 months post inciting event; AC, anticonvulsant; TCA, tricyclic antidepressant; SNRI, serotonin–norepinephrine reuptake inhibitor; NSAID, non-steroidal anti-inflammatory drug; opioids-II, WHO-class II opioid (e.g., Tramadol); opioids-III, WHO-class III opioid (e.g., Tapentadol)*.

*^a^Assessed on the numeric rating scale, 0–10*.

*^b^Stimulation for only 3 days*.

*^c^Use of self-adhesive electrodes for stimulation instead of the hand pad*.

*^d^CRPS type II with lesion of the superficial branch of the radial nerve innervating the dorsum of the hand*.

### Study Design

In this non-randomized open pilot study, 16 of the 20 CRPS patients were stimulated with high-frequency electrical pulses (HF-rSS). The application of the LF-rSS protocol was terminated after four patients, as impairment of the primary outcome parameter (2PD) was observed.

Two-point-discrimination threshold (2PDTH) as primary outcome measure and additionally MDT as a second component of tactile performance as well as current pain intensity were assessed immediately before the start of the intervention and 1–4 h after the termination of the last rSS application.

### Repetitive Sensory Stimulation

Repetitive sensory stimulation was applied on five consecutive days. Each single session lasted 45 min. Electrical stimuli were generated by a two-channel stimulation-device (ELPHA II 3000, danmeter, Denmark) and were conveyed to the fingertips of all fingers of the affected hand by a custom-made hand pad. In case of one patient (patient 4, Table 1), stimulation was terminated after only 3 days. This patient reported that the rigid posture of the fingers on the hand pad lead to a feeling of discomfort, although the electric stimulation itself was not experienced as painful. In one patient, stimuli were transmitted via small (1 cm × 4 cm) self- adhesive electrodes taped to the first and the third phalanx of each finger. Self-adhesive electrodes were connected to the stimulation-device by two leads. In this patient (patient 10, Table 1), the hand pad could not be used due to a 90° flexed position of D5 which impeded contact to the pad. The high-frequency stimulation sequence (HF-rSS) consisted of bursts of 1 s (single pulse duration: 0.2 ms (square), ramp/fall time: 0.5 s, frequency: 20 Hz) with an interburst interval of 5 s. In the low-frequency stimulation protocol (LF-rSS), single pulses of 0.2 ms duration were delivered with 1 Hz. The stimulation intensities for the digits 1–3 (median nerve) and for digits 4 and 5 (ulnar nerve) were controlled separately by two channels. Stimulation intensity was adjusted individually until the patient reported a distinct prickling sensation, while care was taken not to elicit any painful sensation. Mean stimulus intensity was 6.9 ± 2.8 mA (median nerve) and 4.0 ± 1.7 mA (ulnar nerve) for the HF-rSS and 6.6 ± 2.0 mA (median nerve) and 4.9 ± 1.6 mA (ulnar nerve) for the LF-rSS.

### Two-Point-Discrimination

Tactile 2PD of the index finger of the affected and unaffected hand was assessed using a method of constant stimuli, as described previously (20, 24). We used a custom-made device consisting of a rotatable disc with needle probes and an armrest. Seven pairs of rounded needle probes were installed (diameter: 200 μm) with separation distances between 1 and 4 mm (1.0, 1.4, 1.8, 2.2, 2.6, 3.2, and 4 mm). As a control measure zero distance was tested with only a single-needle probe. In case, the patient was not able to reliably discriminate the probes with the largest separation of 3.2 and 4 mm, a second rotatable disc with separation distances of 1.5, 2.3, 3.1, 3.9, 4.7, 5.6, and 7 mm was used. The rotatable disc allows switching rapidly between the probes. To accomplish a uniform and standardized stimulation, the disc was installed in front of an armrest that was moved up and down by the examiner. The test finger was held in a hollow containing a small hole through which the distal phalanx of the index finger was allowed to touch the probes approximately at the same indentations (about 0.5 mm) in each trial. After one demonstration session to become familiar with the task, the subject was strictly instructed not to move the fingertip during the following two test sessions. The seven-needle pairs with different distances and the single needle were presented seven times in randomized order resulting in 56 single trials per session. Subjects were aware that in some trials, single needle probes were presented, but they did not know their frequency of occurrence. The patient had to decide immediately if he had the sensation of one or two tips by answering “one” or “two.” In case of doubt, for instance when the subject reported to have the impression that the stimulus feels “somehow broader than one stimulus,” the subjects were instructed to report “one” as well.

### Mechanical Detection Threshold

The MDT was assessed bilaterally on the palmar tip of the index finger using a standardized set of modified von Frey filaments (VF1 OptiHair_2_, Marstocknervtest, Marburg, Germany) exerting forces between 0.25 and 512 mN in five ascending and descending stimulus intensities. The final threshold was calculated as the geometric mean of the resulting five suprathreshold and five subthreshold values.

### Assessment of Pain Intensity

Patients were asked to rate their current pain intensity at rest on an 11-point numerical rating scale (NRS 0–10), with NRS = 0 meaning no pain and NRS = 10 meaning the worst pain imaginable.

### Statistical Analyses

All data are expressed as mean ± SEM. In case of the calculation of the mean current pain intensity, the data of patients who rated 0 for no pain (*n* = 2) were not included. For calculation of the 2PDTH, the summed responses were plotted against distance as a psychometric function for the absolute threshold and were fitted by a binary logistic regression (SPSS^®^, Version 21.0, IBM, Ehningen, Germany). Threshold criterion was 50% correct responses. After testing for normal distribution (Kolmogorov–Smirnov test), differences in 2PDTH, MDT, and current pain intensity before and after treatment were assessed by the Wilcoxon matched pairs test. Differences between affected and non-affected side were analyzed by the Mann–Whitney *U* test. For correlation analyses, Pearson’s partial correlation corrected for the factor age was calculated. Statistical significance was assumed if *p* < 0.05.

## Results

### High-Frequency Repetitive Sensory Stimulation

#### Two-Point-Discrimination-Thresholds

**Figure 1:**
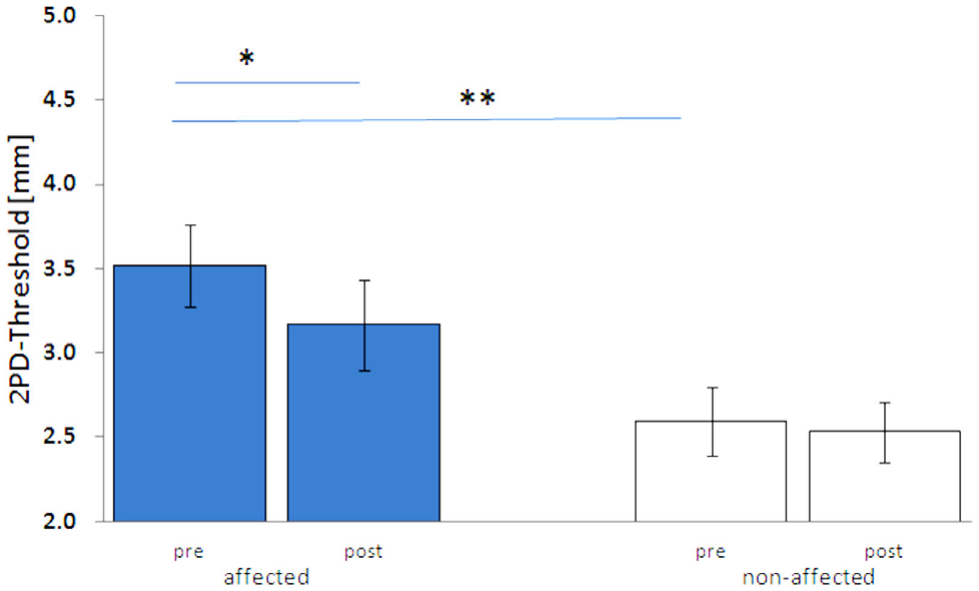
**Two-point-discrimination thresholds (2PDTH, mean ± SE) of the affected and non-affected hand pre and post HF-rSS**. **p < 0.01.

Before treatment, the mean 2PDTH of the index finger of the affected hand was significantly higher than on the non-affected hand (3.52 ± 0.25 vs. 2.59 ± 0.21 mm, *p* < 0.01). Discrimination thresholds of the non-affected hand were in the same range as those reported for healthy age-matched controls (25). After 5 days of HF-rSS, the 2PDTH on the affected hand significantly improved, as shown by lowered 2PDTH (3.17 ± 0.27 mm, *p* < 0.01, Figure 1). On average, the discrimination threshold was improved by 10.4 ± 3.6% on the affected hand. On the non-affected hand, the 2PDTH did not change (2.53 ± 0.18 mm, *p* = 0.50, Figure 1).

#### Mechanical Detection Threshold

**Figure 2:**
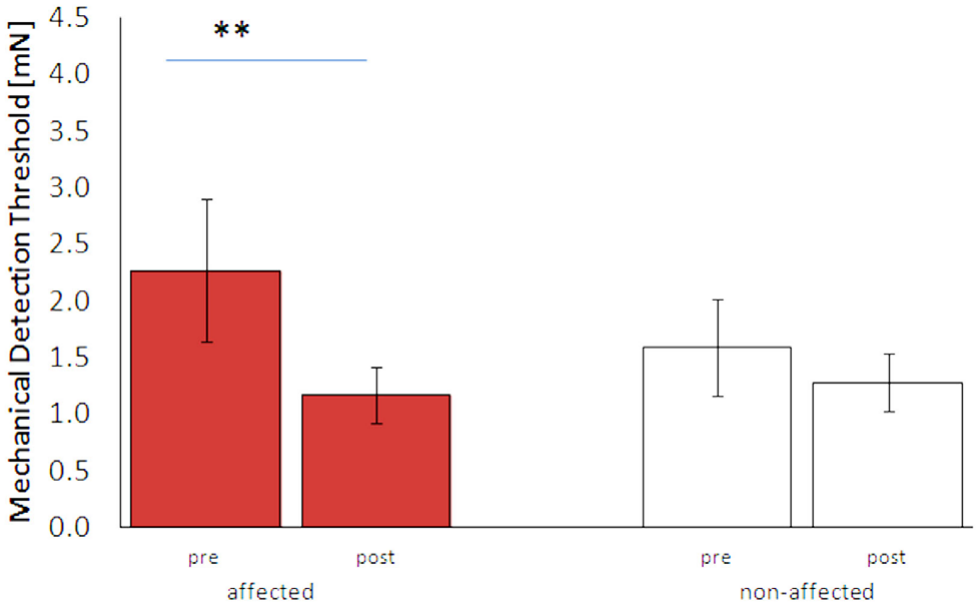
**Mechanical detection thresholds (MDT, mean ± SE) of the affected and non-affected hand pre and post HF-rSS**. **p < 0.01.

Before the intervention, the mean MDT of the affected hand was higher than on the non-affected hand (2.26 ± 0.63 vs. 1.59 ± 0.43 mN, *p* = 0.28), though not being statistically significant. However, MDT of the non-affected hand was substantially higher than those observed for healthy age-matched controls (unpublished). After the treatment, the mean MDT decreased significantly on the affected hand (1.17 ± 0.25 mN, *p* < 0.01, Figure 2). Mean gain in performance was 28.4 ± 18.4%. On the non-affected hand, the MDT did not significantly change, although there was a clear trend toward lower MDTs (1.28 ± 0.26 mN, *p* = 0.25).

#### Pain

The mean level of current pain at rest before the start of the treatment was 3.8 ± 0.49. The mean pain intensity after 5 days of stimulation was 3.4 ± 0.52. Differences in pain intensity before and after the intervention were not statistically significant. However, four patients showed a reduction in pain of at least 30% (Table 1).

#### Correlation Analyses

Two-point-discrimination threshold before and after the intervention, MDT before and after the intervention, as well as current pain intensity before and after the intervention were significantly correlated (2PDTH: *r* = 0.84, *p* < 0.01; MDT: *r* = 0.69, *p* < 0.01, pain: *r* = 0.65, *p* < 0.05). Neither before nor after the intervention performances in both tactile tests were related to each other or to the factor pain. Concerning the percentage of change of both tactile measures and the change of pain intensity, there was no significant correlation between all measures (Table 2).

**Table 2:** **Partial correlation coefficients of tactile performance parameters and pain intensity, with age as control variable**.

Parameter	Disease duration	2PDTH affected hand, pre	2PDTH affected hand, post	Δ 2PDTH affected hand	MDT affected hand, pre	MDT affected hand, post	Δ MDT affected hand	Current pain, pre	Current pain, post	Δ current pain
Disease duration		0.219	−0.146	−0.512	−0.086	0.477	0.733	0.463	0.429	−0.028
2PDTH affected hand, pre	0.219		0.840	0.082	−0.161	0.224	0.465	0.276	−0.065	−0.323
2PDTH affected hand, post	−0.146	0.840**		0.597	0.133	0.214	0.048	0.203	−0.091	−0.263
Δ 2PDTH affected hand	−0.512	0.082	0.597		0.558	0.182	−0.505	0.009	−0.027	−0.010
MDT affected hand, pre	−0.086	−0.161	0.133	0.558		0.695	−0.257	0.118	−0.112	−0.373
MDT affected hand, post	0.477	0.224	0.214	0.182	0.695**		0.439	0.326	−0.031	−0.513
Δ MDT affected hand	0.026	0.465	0.048	−0.505	−0.257	0.439		0.147	0.106	−0.077
Current pain, pre	0.463	0.276	0.203	0.009	0.118	0.326	0.147		0.653	−0.307
Current pain, post	0.429	−0.065	−0.091	−0.027	−0.112	−0.031	0.106	0.653*		−0.495
Δ Current pain	−0.028	−0.323	−0.263	−0.010	−0.373	−0.513	−0.077	−0.307	0.495	

*2PDTH, two-point-discrimination threshold; MDT, mechanical detection threshold*.

*Δ, % change (pre vs. post)*.

Bold font indicates significant p values

**p < 0.01, *p < 0.05.

#### Low-Frequency Repetitive Sensory Stimulation

**Figure 3:**
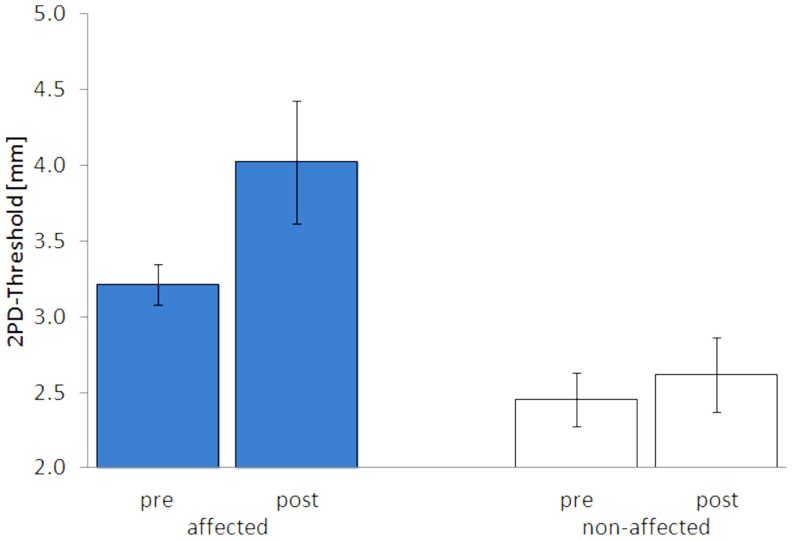
**Two-point-discrimination thresholds (2PDTH, mean ± SE) of the affected and non-affected hand pre and post LF-rSS**.

**Figure 4:**
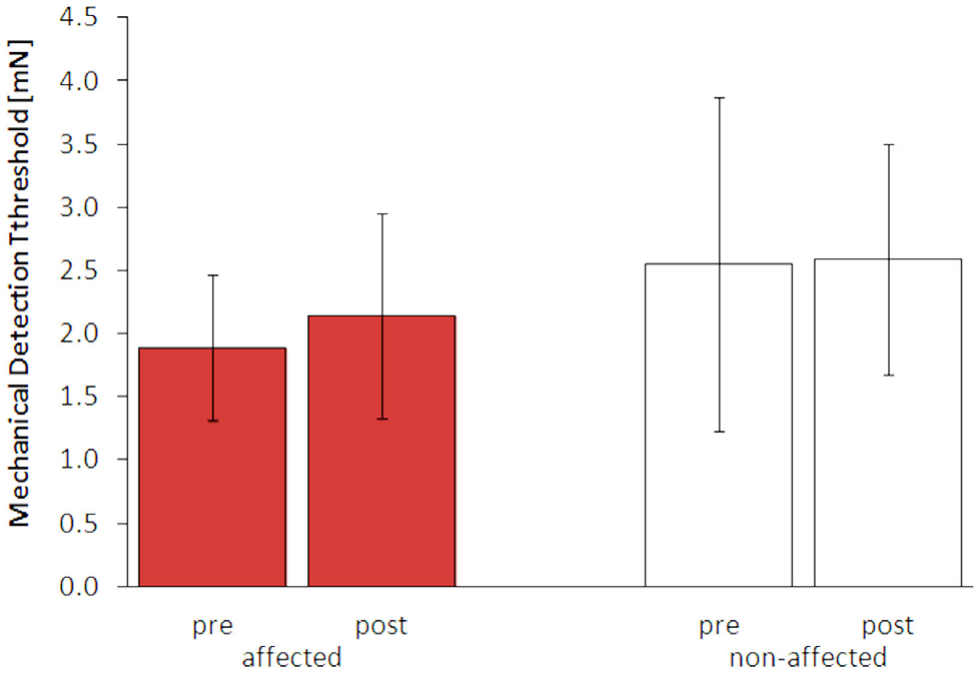
**Mechanical detection thresholds (MDT, mean ± SE) of the affected and non-affected hand pre and post LF-rSS**.

After 5 days of LF-rSS, performance in 2PD on the affected hand was impaired in all four patients (Figure 3, range: 9.3–71.4%, 3.2 ± 0.13 vs. 4.0 ± 0.40 mm). Mean 2PDTH on the non-affected hand were comparable before and after treatment (range: −1.6–11.3%, 2.45 ± 0.18 vs. 2.61 ± 0.25 mm). The MDT was not affected consistently by the treatment among the patients (Table S1 in Supplementary Material): two patients had lowered thresholds (−55 and −15%), one patient had an elevated MDT (110%), and one patient’s MDT was unchanged. Mean MDT before and after treatment are shown in Figures 3 and 4 (affected: 1.89 ± 0.57 vs. 2.15 ± 0.81 mN; non-affected: 2.55 ± 1.32 vs. 2.59 ± 0.92 mN). None of the patients showed pain relief by 30% or more post LF-rSS (Table S1 in Supplementary Material; 4.3 ± 1.50 vs. 4.3 ± 0.6).

## Discussion

After application of HF-rSS to the affected hand of CRPS patients on five consecutive days, performance in 2PD and mechanical detection significantly improved on the affected hand. The average gain in performance was about 10% for the 2PDTH and about 28% for the MDT. The mean current pain intensity was not reduced after HF-rSS, although individual patients showed a pain relief of 30% or more. The few patients receiving LF-rSS showed further deteriorated 2PD on the affected hand.

### Improvement of 2PD Performance – Relation to Other Studies

Following HF-rSS, tactile acuity as measured by 2PD was significantly improved on the affected hand with an average gain in performance of about 10%. In other studies applying HF-rSS in healthy subjects, the gain in tactile acuity was in the range of approximately 10–20%, depending on whether one single digit or all digits of the hand were stimulated and whether HFS-rSS was applied for one single session or for longer periods (24, 25, 40–42). Some years ago, the so-called tactile coactivation protocol adopted from earlier studies by Godde et al. (20) had been applied in a single 3-h session to one digit in CRPS patients and to healthy controls (43) to study the perceptual learning ability in CRPS patients. For patients, they reported an average gain in tactile acuity of approximately 7%, which was significantly lower compared to the healthy controls (16%). This study suggested that coactivation is in principle effective in CRPS patients by inducing improvement of tactile acuity, however, to a lesser extent. Considering the data of the present investigation, large interindividual differences among the patients were revealed. For instance, in three patients 2PD threshold changes were <5%, but in five patients we measured improvements of about 20–30% (Table 2). One possible explanation might have been differences in the initial performance of each individual. Kalisch and coworkers, for instance, (40) reported a baseline-dependency of the stimulation effect after application of HF-rSS, meaning the individuals with the highest initial discrimination thresholds had the largest gain in performance. Correlation analyses of our data revealed no such baseline-dependency (Table 2). Thus, in CRPS patients other factors than the initial performance impairment must be responsible for the different magnitudes in performance gain. However, correlation analyses of various factors that might be related to the individual performance gain in the present study, as for instance current pain intensity or MDT, revealed no variables accountable for the interindividual differences. It is noteworthy that all patients in the present study during the period of rSS also received standard occupational- and physio-therapy, making it difficult to differentiate between the impacts of both therapies. Other studies performed in healthy individuals, or in stroke patients demonstrated that the strength and also the persistence of the stimulation effects can be increased further by extending the period of daily stimulation sessions (28, 40). In case of chronic stroke patients, the time needed to induce stimulation effects could be several weeks or months (28). Conceivably, CRPS patients with only little gain in tactile performance, or little pain reduction might need longer periods than 5 days of stimulation to generate stable effects.

### Effects of Low-Frequency-rSS

In all of the four patients stimulated with LF-rSS, impaired 2PD performance was detected. This is in line with findings in healthy individuals after LF-rSS (26) stimulation and corroborates the hypothesis that rSS is related to LTP-, respectively, LTD-like processes. The effects induced by LF-rSS and HF-rSS were qualitatively similar in CRPS patients and in healthy subjects, indicating integrity of cortical capacity for processing rSS stimuli in CRPS. However, these findings clearly indicate that a low-frequency stimulation protocol is unsuitable for further studies using rSS in CRPS patients.

### Cortical Involvement in CRPS

From imaging as well as from studies measuring somatosensory evoked potentials in healthy persons (21–23) and in CRPS patients (9, 12, 29–33), it is known that changes of performance in tactile discrimination are linked to cortical map extension of the respective body part, so that 2PDTH can be used as surrogate marker for cortical map changes. Thus, the present data can be interpreted as a hint of cortical map enlargement of the affected hand in the CRPS patients following HF-rSS. Apparently, the impact of the rSS stimuli on 2PD performance is comparable in healthy persons and in CRPS patients, i.e., improvement of the 2PD following HF-rSS and impairment following LF-rSS (see below). Hence, despite disorganization of somatosensory maps in CRPS our data provide evidence that rSS stimuli might be processed similarly in healthy individuals and in CRPS patients.

### Pain Intensity

The average current pain intensity of CRPS patients was not affected by the HF-rSS treatment; however, in four patients, pain decreased by at least 30%. Since studies from amputees with phantom limb pain (11, 44, 45) and also from CRPS patients (9) report improved tactile discrimination performance after sensory training in combination with cortical representational changes in SI and decreased pain intensity, it is reasonable to hypothesize that HF-rSS, which is known to improve tactile function and cortical organization, will also decrease pain in CRPS patients. Nevertheless, on average, the HF-rSS application over 5 days resulted in only limited pain reduction. Despite the lack in correlation between pain measures and markers of tactile performance (Table 2), we compared the four patients with pain reduction with the 12 patients who did not have pain reduction concerning their disease duration, severity, pain medication usage, and HF-rSS stimulation intensity. We found that none of these variables differed between those with or without pain reduction. The only conspicuous feature was that three of the four patients with pain reduction had very high MDTs. However, because of the rather limited sample size these observations have to be verified in upcoming studies. One plausible explanation is that the stimulation duration of 5 days was too short to influence pain intensity. Thus, further studies with extended periods of daily stimulation sessions are needed. In addition, the small sample size made it hard to draw conclusions from the data of the patients that showed pain reduction, which requires larger patient groups. Finally, further studies will need controls using sham stimulation to provide further evidence about the interventional potential of rSS in CRPS patients not only in respect to recovered tactile discrimination abilities but also concerning pain relief. Another possible explanation for the lack of impact on current pain intensity might be that pain is processed in cortical regions not affected by HF-rSS. Recent investigations on cortical foci of pain processing however provide conflicting results. For example, Mancini et al. (8, 46) reported that the fingertips have overlapping cortical maps of tactile and nociceptive inputs, supporting the assumption that HF-rSS could affect cortical pain processing.

### Eligibility as Additional Therapeutic Approach

In all but two patients rSS applied by means of the hand pad was well feasible. In one patient, the hand pad was not suitable, because one finger could not contact the stimulation area for this finger on the pad due to a flexion contracture. In this case, we used adhesive electrodes to transmit the stimuli. In a second patient, we terminated stimulation with the hand pad after 3 days because this patient experienced the rigid position of the fingers during the third session as unpleasant. None of the patients reported the electric stimulation itself to be painful or unpleasant. Thus, HF-rSS with the stimulation device used in this study is suitable for the use in patients with CRPS of the upper limb. In rare cases where no sufficient contact of all fingers with the stimulation area is possible, adhesive electrodes could be used alternatively to apply HF-rSS pulses. Since an intact somatosensory input is essential for recovery of handfunction (47–49), the positive impact of HF-rSS on tactile performance makes it a promising new candidate as additional therapeutic approach in the rehabilitation of the affected limb.

## Conclusion

Our results demonstrate a good feasibility and efficacy of HF-rSS in CRPS patients to improve sensory loss, therefore providing an essential prerequisite for rehabilitation of handfunction. Qualitatively, stimulation effects were similar to those observed in healthy controls, indicating intact cortical processing of rSS stimuli in patients with CRPS type I. The limited impact on pain relief might be due to the short stimulation period. In order to further evaluate this, studies with an extended period of daily stimulation are needed.

## Conflict of Interest Statement

Marianne David, Hubert R. Dinse, Martin Tegenthoff and Christoph Maier hold patents about repetitive sensory stimulation applications. The remaining authors have no conflict of interest to declare.
