# Supplementary material for: High-Frequency Repetitive Sensory Stimulation as Intervention to Improve Sensory Loss in Patients with Complex Regional Pain Syndrome I
Source: Front Neurol. 2015 Nov 17;6:242. doi: 10.3389/fneur.2015.00242 (PMC4648023; doi:10.3389/fneur.2015.00242)
Supplement: Supplementary file 1 [file Table_1.DOCX]

**Supplementary** **Table 1.** Individual results

a. Stimulation with high frequency pulses (HF-rSS)

| Patient | Current pain, pre (NRS, 0-10) | Current pain, post (NRS, 0-10) | Δ current pain (%) | 2PDTH affected hand, pre (mm) | 2PDTH affected hand, post (mm) | Δ 2PDTH affected hand (%) | 2PDTH non-affected hand, pre (mm) | 2PDTH non-affected hand, post (mm) | Δ 2PDTH non-affected hand (%) | MDT affected hand, pre (mm) | MDT affected hand, post (mm) | Δ MDT affected hand (%) | MDT non-affected hand, pre (mm) | MDT non-affected hand, post (mm) | Δ MDT non-affected hand (%) |
| --- | --- | --- | --- | --- | --- | --- | --- | --- | --- | --- | --- | --- | --- | --- | --- |
| 1 | 7 | 7 | 0 | 2.8 | 2.7 | -4 | 2.0 | 2.2 | 9.5 | 2.2 | 1.5 | -30.2 | 0.8 | 0.3 | -65.6 |
| 2 | 0 | 0 | - | 3.1 | 2.3 | -25 | 2.7 | 2.4 | -11.0 | 0.3 | 0.3 | 0.0 | 0.2 | 0.5 | 116.7 |
| 3 | 5 | 0 | -100 | 4.3 | 3.9 | -10 | 3.8 | 3.3 | -12.4 | 3.7 | 2.6 | -28.8 | 1.8 | 1.4 | -24.7 |
| 4 | 3 | 4 | 33 | 4.5 | 4.6 | 2 | 2.0 | 1.9 | -4.9 | 0.5 | 0.2 | -56.8 | 2.9 | 2.7 | -6.9 |
| 5 | 3 | 2 | -33 | 2.3 | 2.3 | 1 | 2.2 | 2.4 | 11.9 | 9.6 | 2.9 | -69.8 | 6.0 | 2.2 | -63.3 |
| 6 | 3 | 6 | 100 | 2.4 | 2.3 | -6 | 1.8 | 1.8 | 4.0 | 0.9 | 0.3 | -72.2 | 0.2 | 0.7 | 268.4 |
| 7 | 5 | 6 | 20 | 3.0 | 2.2 | -26 | 1.9 | 2.2 | 15.6 | 2.2 | 0.7 | -68.4 | 1.4 | 1.5 | 9.3 |
| 8 | 1 | 1 | 0 | 3.2 | 2.8 | -14 | 2.4 | 2.8 | 16.7 | 1.1 | 1.1 | 0.0 | 0.8 | 0.8 | 0.0 |
| 9 | 1 | 1 | 0 | 4.8 | 4.5 | -6 | 5.0 | 4.5 | -10.2 | 1.0 | 0.5 | -53.8 | 1.2 | 2.9 | 135.8 |
| 10 | 4 | 4 | 0 | 2.7 | 2.0 | -26 | 2.5 | 2.2 | -15.0 | 1.3 | 1.2 | -9.6 | 1.3 | 1.2 | -2.0 |
| 11 | 7 | 4 | -43 | 4.8 | 4.3 | -10 | 3.2 | 3.7 | 16.2 | 0.5 | 0.4 | -28.6 | 0.2 | 0.2 | 0.0 |
| 12 | 6 | 4 | -33 | 4.0 | 3.7 | -7 | 2.3 | 2.2 | -3.0 | 4.2 | 1.5 | -64.3 | 3.1 | 2.4 | -22.6 |
| 13 | 5 | 5 | 0 | 5.3 | 3.6 | -32 | 2.9 | 2.2 | -23.3 | 0.9 | 2.9 | 229.5 | 4.4 | 2.9 | -34.1 |
| 14 | 3 | 3 | 0 | 4.1 | 5.2 | 27 | 2.3 | 2.1 | -7.0 | 6.0 | 2.2 | -63.3 | 0.2 | 0.2 | 0.0 |
| 15 | 2 | 2 | 0 | 2.6 | 2.4 | -8 | 2.5 | 2.4 | -4.0 | 1.5 | 0.5 | -70.0 | 0.8 | 0.5 | -40.0 |
| 16 | 2 | 2 | 0 | 2.7 | 2.1 | -22 | 2.0 | 2.1 | 3.5 | 0.6 | 0.2 | -68.3 | 0.2 | 0.2 | 0.0 |
| Mean ± SE | 3.8±0.5 | 3.4±0.5 | -3.8±10.6 | 3.5±0.3 | 3.2±0.3 | -10.6±3.6 | 2.6±0.2 | 2.5±0.2 | -0.8±3.1 | 2.3±0.6 | 1.2±0.3 | -28.4±18.4 | 1.6±0.4 | 1.3±0.3 | 16.9±21.6 |

b. Pilot testing with low frequency pulses (LF-rSS)

| Patient | Current pain, pre (NRS, 0-10) | Current pain, post (NRS, 0-10) | Δ current pain (%) | 2PDTH affected hand, pre (mm) | 2PDTH affected hand, post (mm) | Δ 2PDTH affected hand (%) | 2PDTH non-affected hand, pre (mm) | 2PDTH non-affected hand, post (mm) | Δ 2PDTH non-affected hand (%) | MDT affected hand, pre (mm) | MDT affected hand, post (mm) | Δ MDT affected hand (%) | MDT non-affected hand, pre (mm) | MDT non-affected hand, post (mm) | Δ MDT non-affected hand (%) |
| --- | --- | --- | --- | --- | --- | --- | --- | --- | --- | --- | --- | --- | --- | --- | --- |
| 17 | 6 | 5 | -17 | 3.60 | 4.01 | 11.4 | 2.13 | 2.245 | 5.4 | 0.5 | 0.2 | -55.6 | 1.5 | 5.0 | 233.3 |
| 18 | 4 | 4 | 0 | 3.18 | 3.56 | 11.9 | 2.78 | 3.095 | 11.3 | 2.6 | 2.2 | -15.4 | 2.5 | 2.9 | 16.0 |
| 19 | 3 | 4 | 33 | 3.07 | 3.355 | 9.3 | 2.17 | 2.135 | -1.6 | 3.0 | 6.3 | 110.0 | 4.2 | 1.7 | -59.5 |
| 20 | 0 | 0 | - | 3.01 | 5.16 | 71.4 | 2.73 | 2.98 | 9.2 | 1.5 | 1.5 | 0.0 | 0.4 | 0.8 | 97.4 |
| Mean ± SE | 4.3±0.3 | 4.3±0.3 | 5.6±12.7 | 3.2±0.1 | 4.0±0.4 | 25.1±15.2 | 2.5±0.2 | 2.6±0.3 | 6.1±2.9 | 1.9±0.6 | 2.6±1.3 | 9.8±35.4 | 2.2±0.8 | 2.6±0.9 | 71.8±62.7 |

Abbreviations: NRS = Numeric Rating Scale, 2PDTH = 2-Point-Discrimination Threshold, MDT = Mechanical Detection Threshold
